# Photosynthetic Properties of Co-Occurring Pioneer Species on Volcanically Devastated Sites in Miyake-jima Island, Japan

**DOI:** 10.3390/plants10112500

**Published:** 2021-11-18

**Authors:** Xiulong Zhang, Hao Li, Xiaoxing Hu, Pengyao Zheng, Mitsuru Hirota, Takashi Kamijo

**Affiliations:** 1 Graduate School of Life and Environmental Sciences, University of Tsukuba, Tsukuba 305-8572, Japan; zhangxl1@cib.ac.cn (X.Z.); s1921192@s.tsukuba.ac.jp (P.Z.); 2 Eco-Environment Design and Research Institute, China Railway Eryuan Engineering Group Co. Ltd., Chengdu 610031, China; leehboy@hotmail.com; 3 College of Biology and Agricultural Resources, Huanggang Normal University, Huanggang 438000, China; huxiaoxing@hgnu.edu.cn; 4 Faculty of Life and Environmental Sciences, University of Tsukuba, 1-1-1 Tennoudai, Tsukuba 305-8572, Japan; hirota0313@gmail.com

**Keywords:** volcanically devastated site, pioneer species, ecophysiological leaf traits, photosynthetic N use efficiency

## Abstract

Pioneer species differing in their inherent ecological characteristics (e.g., N-fixing ability, photosynthetic pathway) can have a large impact on local ecosystems in the early stages of volcanic succession. However, it remains unclear as to how these pioneer species adapt to the extreme environment of volcanically devastated sites in terms of ecophysiological leaf traits. In this study, we compared the leaf traits (including morphological, physiological) of three co-occurring pioneer species, including a C_4_ non-N-fixing grass, a C_3_ N-fixing tree, and a C_3_ non-N-fixing herb from a newly created (18 years after eruption) volcanically devastated site in Miyake-jima, Japan. Our results showed that three pioneer species have different sets of leaf traits that are associated with their ecophysiological growth advantages, respectively. *Miscanthus condensatus* shows the highest light-saturated photosynthetic rate (A_max_). The higher A_max_ were partially the result of higher water use efficiency (WUE) and photosynthetic N-use efficiency (PNUE). The PNUE in *M. condensatus* appears to be high, even for a C_4_ grass. *Alnus sieboldiana* rely on its N-fixing ability, has a higher leaf N content (N_area_) that compensates for its photosynthetic machinery (Rubisco), and further ensures its photosynthetic capacity. *Fallopia japonica* var. *hachidyoensis* has a higher leaf mass per area (LMA), chlorophyll content (Chl), and maximum quantum yield of PSII (Fv/Fm), demonstrating its higher light capturing ability. These results make it possible to predict certain ecological processes that take place in the early stages of volcanic succession resulting from ecological characteristics and from some key leaf traits of pioneer species. It also provides a theoretical basis for species selection and species combination for volcanic ecological restoration.

## 1. Introduction

Volcanic eruptions are major natural disturbances with varied and complex consequences [1]. Among the nutritional constraints, nitrogen (N) limitation largely impedes plant growth on the early stages of volcanic deposits [2,3]; only a limited number of pioneer species can adapt to harsh volcanic environments due to their inherent ecological characteristics (e.g., C_4_ photosynthesis pathway with higher CO_2_ utilization rate and lower water demand and N-fixing ability). Pioneer species generally have certain characteristics that allow them to grow and persist in harsh condition. Several studies that have clarified the success pioneer species under harsh volcanic environments have focused on vegetation (growth form), life history (dormancy form), phenology, seed biology [4], biomorphology (e.g., root architecture, shoot shape), and seed viability (seed size/mass) [5,6]. However, from the perspective of photosynthetic-related leaf traits (including morphological, physiological), which are closely related to plant fitness and survival strategies [7] and are essential for the successful maintenance and expansion of populations, have received little attention [8,9,10,11].

The leaf traits that are associated with plant growth strategies are consistently affected by the ecological characteristics of a species, such as the photosynthetic pathway (C_3_ or C_4_) [12] or the N-fixing ability [13,14]. Many studies have focused on leaf traits in relation to the ecological characteristics of a species [15,16,17,18]. Some studies have shown significant overall leaf trait differences between species that differ with respect to the photosynthetic pathway and/or N-fixing ability [19,20,21]; nevertheless, the magnitude of these differences is influenced by environmental conditions and the plant functional group [19,20,21,22]. N-fixing ability is one of the key ecological characteristics that cause a plant to be able to successfully colonize and establish itself, especially in N-poor ecosystems. The colonization of new surfaces by N-fixing plant species results in the greatly improved soil fertility, ecosystem process rates, and nutrition of co-occurring non-N-fixing plant species [23,24,25]. Further, N-fixing species often have resource conservative strategies that can be associated with higher N concentrations (N_area_) in the their leaves [13,26], a lower leaf mass per area (LMA) [27,28], and lower photosynthetic N use efficiency (PNUE) [29], as these species have continuous N input that can be achieved through biological N fixation ability when compared to non-N-fixing species; again, these traits could lead to large ecosystem-level consequences. For those species without N-fixing ability but that are differing in terms if the photosynthetic pathway, there is a general suite of leaf trait divergences between C_3_ and C_4_ species, with most evidence coming from the Poaceae family, the family from which C_4_ photosynthesis has evolved from multiple times independently [30]. Across a sample of 382 C_3_ and C_4_ grasses, Atkinson et al. [31] found that LMA was significantly higher in C_4_ species than it was in C_3_ species. Together, these traits enable greater energy capture for a given leaf mass investment [28,32], and in combination with the higher photosynthetic efficiencies of different C_4_ species, led to a growth advantage of C_4_ species [31]. In addition, the C_4_ CO_2_-concentrating mechanism also ensures that the C_4_ species fix more carbon for a given investment in photosynthetic proteins, leading to higher nitrogen-use efficiency and, as a consequence, a lower leaf nitrogen requirement [33,34,35,36], ensuring their success. However, to our knowledge, the PNUE differences of C_3_, C_4_, and N-fixer that co-occur in a volcanic N-deficient habitat have not been directly compared. Furthermore, if the key leaf traits of co-occurring pioneer species do indeed differ from the N-fixing ability and photosynthetic pathway under a volcanic N-deficiency habitat, then the impacts of plant species on ecological processes such as productivity could potentially be predicted from their photosynthetic pathway and N fixation ability.

To understand the variation in the key leaf traits that are associated with the ecological strategies of volcanic pioneer species differing in N-fixing ability and in the photosynthetic pathway, we compared the leaf traits for three co-occurring pioneer species in an early successional volcanic system in Miyake-jima Island, Japan. *Alnus sieboldiana* (a C_3_ N-fixing tree), *Miscanthus condensatus* (a perennial C_4_ grass), and *Fallopia japonica* var. *hachidyoensis* (a perennial C_3_ herb) are common pioneer species that co-occur on a volcanically devastated site in Miyake-jima [37,38]. Among these three species, *A. sieboldiana* (with N-fixing ability) can partially grow in volcanic N-deficiency deserts, as it has the ability to fix N in the atmosphere via symbiosis with nitrogen-fixers. Additionally, Choi et al. [39] also proved its success in resisting high concentrations of volcanic gas (SO_2_) in the Miyake-jima volcano. Regarding *M. condensatus*, our previous study [11] in the volcanically devastated site in Miyake-jima indicated that the relatively high light-saturated photosynthetic rate (A_max_) and PNUE of *M. condensatus* were its adaptation advantages to volcanically N-deplete habitats. As for *F. japonica*, Sakata et al. (2006) [40] indicated that at the higher activation state, the Rubisco in Aconogonum weyrichii (closely related species of *F. japonica*) can also be regarded as an adaptive feature in summer, as it allows for intensive dry-matter production within the short growing period. Although these three species are studied individually in different habitats, we know very little about their physiological response to a sole harsh volcanic habitat with multi adverse factors. Further, to the best of our knowledge, there almost no comparative studies of the PNUE of pioneer species on single volcanically devastated site. Likewise, this study provides some evidence to study ecophysiological patterns compared to those that are known for other similar N-limited habitats, those in in Japan [40], Hawaii [20,41], Australia [42], and New Zealand [43], for example.

Based on the information above, we described plant strategies using 12 key leaf traits to test the following hypotheses: 

(1) Leaf traits associated with carbon capture strategies are consistently affected and by N fixation ability [13,26]. Additionally, as N would be the primary limiting factor in a volcanically devastated site, we expected that the N-fixing pioneer species would show leaf traits that are related to conservative strategies (higher N_area_ with relatively low A_max_, PNUE) [13,26,29], while the non-N-fixing pioneer species should have opposite strategies.

(2) Between two non-N-fixing species, *M. condensatus* should have more resource-acquisitive leaves than *F. japonica*, showing lower LMA values but higher PNUE and water use efficiency (WUE) values in *M. condensatus*.

In testing our hypotheses, the specific goals were to (1) document the lead trait values of three pioneer species in the early stages of a volcanically devastated site and to (2) examine whether the leaf trait relationships varied among three pioneer species.

## 2. Results

### 2.1. Growth Condition of Three Pioneer Species

Table 1 shows the growth conditions of each species during the entirety of the measurement period. There were almost no differences in canopy openness (CO), precipitation, or air temperature (AT) at each species (Table 1). The total C content (STC) and N content (STN) in the soil under the *A. sieboldiana* and *F. japonica* individuals were significantly higher than the total C content found under *M. condensatus* (Table 1). All of the individuals were measured in newly volcanically devastated site.

### 2.2. Species Difference in Leaf Traits

Table 2 and Table 3 show the measured leaf traits that are associated with each traits’ function as well as their mean values of each pioneer species. Almost all of the leaf traits (except light compensation point (LCP)) differed significantly (Table 3) among the three species. For the morphological leaf traits, the LA of *M. condensatus* was significantly higher (Table 3) than it was *A. sieboldiana* and *F. japonica*, whereas the LMA of *F. japonica* was significantly higher (Table 3) than it was for the other two species. For the physiological leaf traits, the A_max_, PNUE, and WUE of *M. condensatus* were significantly higher than they were for *A. sieboldiana* and *F. japonica* (Table 3), whereas the transpiration rate (E) was lower. The N_area_ and the dark respiration rate (DR) of *A. sieboldiana* was the highest among the three species (Table 3). For *F. japonica*, the LMA, Fv/Fm, and Chl content were significantly higher than they were *A. sieboldiana* and *F. japonica* (Table 3). We can find that *F*. *japonica* is better at light capture and that *M. condensatus* is better in terms of N use.

### 2.3. Trait’s Relationships of Three Pioneer Species

**Figure 1:**
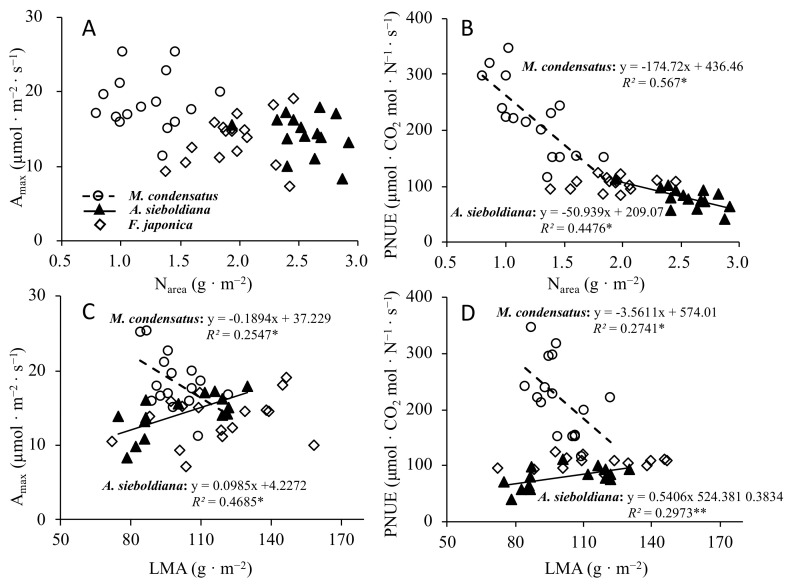
Relationships between leaf nitrogen concentration per leaf area (N_area_) to light-saturated photosynthetic rate (A_max_) (**A**) and photosynthetic N-use efficiency (PNUE) (**B**) and relationships of leaf mass per area (LMA) to A_max_ (**C**) and PNUE (**D**) of three pioneer species on Miyake-jima volcano Island. Symbols: *M. condensatus* (open circles), *A. sieboldiana* (black triangle), *F. japonica* (open rhombus). Significant regression lines: *M. condensatus* (black dashed) and *A. sieboldiana* (solid). Significance of the regression lines: ns *p* > 0.05, * *p* < 0.05, ** *p* < 0.01.

Correlation analyses showed that the N_area_ was not significantly correlated with A_max_ among the three species (Figure 1A). On the other hand, the N_area_ was negatively correlated with the PUNE for *A. sieboldiana* and *M. condensatus* (Figure 1B). LMA was significant positively correlated with A_max_ and PNUE for *A. sieboldiana* but was negatively correlated for *M. condensatus* (Figure 1C). As for *F. japonica*, there were no significant relationships between LMA, A_max_, and PNUE (Figure 1B–D).

## 3. Discussion

### 3.1. Trait Comparison to Previous Studies

Our study first provides some ecophysiological evidence for the success of volcanic pioneer species living at the volcanically devastated habitat in Miyake-jima compared to previous studies. In general, species from N-poor soils had lower leaf N concentrations and photosynthetic capacities than those from N-rich soils. However, we found that the key leaf trait values (N_area_, A_max,_ and PNUE) of three pioneer species were within the normal ranges reported for the C_4_ non-N-fixing grasses, C_3_ non-N-fixing herbs, and C_3_ N-fixing trees worldwide (Glopnet) (Table A1) [28]. The average values of N_area_ for *F. japonica* and A_max_ for *A. sieboldiana* are even higher than the leaf traits from the N-rich habitat (Table A1). This reflects the relatively higher ability of these species to resist the N-low conditions in this volcanic environment; these plants also show an extremely low N requirement and demonstrate more effective N use strategies than the Miyake-jima pioneer species [11,39]. 

To gain further insight into the ecophysiological advantages of Miyake-jima pioneer species, the pioneer species living in similar harsh habitats (e.g., sand dunes, glacier retreated sites, volcanic deserts), for instance, in Hawaii [20,41], Australia [42], New Zealand [43], and Japan [40] were compared, and we found that (1) irrespective of N availability, the photosynthesis ability (A_max_) and N use ability (PNUE) of the C_4_ species are consistently higher than those of the C_3_ plants [12]. Compared to the limited number of other C_4_ pioneer species reported in a volcanically devastated site in Hawaii about 100 years after eruption, the N_area_ of *M. condensatus* is higher, whereas the A_max_ and PNUE are lower [20]. This seems to indicate the unique nitrogen acquisition conservation strategy of *M. condensatus* in extreme N-limited habitats; (2) *F. japonica* also showed relatively high level of N_area_ (second only to *Olearia axillaris* in Australian N-limited sand dunes). Compared to the other C_3_ pioneer herbs, the A_max_ and PNUE are within a normal range [20,40,42]. (3) In terms of C_3_ N-fixing tree species, since very few studies are comparable to our data, only one species (*Coriaria arborea*) reported from a New Zealand glacial retreated habitat [43] showed lower A_max_ and PNUE than *A. sieboldiana*. On the other hand, compared to the other C_3_ non-N-fixing pioneer trees, there were no obvious differences. To a certain extent, the above comparison proved the respective ecophysiological advantages of different pioneer species at the volcanically devastated site in Miyake-jima. 

### 3.2. Interspecific Difference in Leaf Traits-Related Strategies among Three Pioneer Species

**Figure 2:**
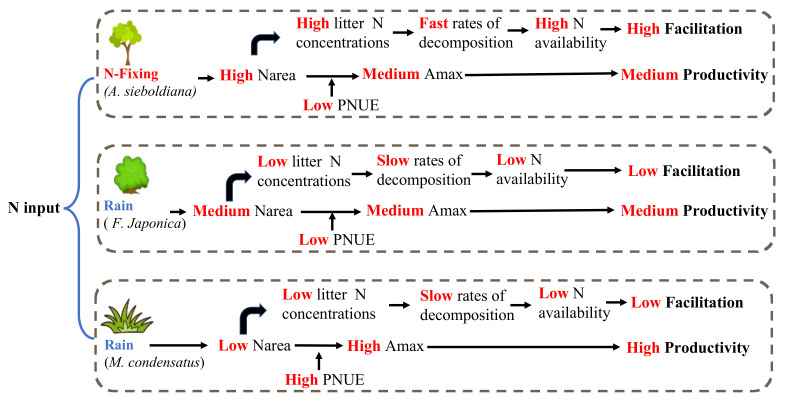
Conceptual figure of the impact of various species on ecosystem functions according to ecophysiological leaf traits.

Leaf traits were closely associated with the growth, survival, and resource (e.g., light and nitrogen) requirements of the species. We found that within the early stage volcanically devastated habitat in Miyake-jima, three coexisting pioneer species indeed differed to a large extent in the suite of their leaf traits (Table 3). The higher photosynthetic capacity and the more efficient water and N use ability (low LMA, N_area_ with high A_max_, PNUE, and WUE) of *M.* *condensatus* than *A. sieboldiana* and *F. japonica* (C_3_ photosynthetic pathway) is easily explained by the fact that C_4_ involves efficient CO_2_ concentrating mechanisms [44]. N-fixer *A. sieboldiana*, as we hypothesized, shows traits that are more closely associated with conservative strategy (higher N_area_ with relatively low A_max_, PNUE and WUE) [13,26,29]) (Table 3). As for *F. japonica*, the higher LMA, Chl, and Fv/Fm values demonstrate its higher ability to intercept and dissipate light [45], and furthermore, it also demonstrates the highest LMA, giving it an advantage in terms of resistance to physical attack, such as strong wind and insect gnawing. Although *A. sieboldiana* and *F. japonica* have the same photosynthetic pathway, there is significant difference in the PNUE between these two species (Table 3), which was first attributed to the relatively low N_area_ of *F. japonica*. Additionally, presumably because of the fundamental differences in the leaf nutrition, this was also the case for the differences in the chemistry and structure between the N-fixing tree and the non-N-fixing herb [14,46]. All of these data indicate that these three pioneer species living in the volcanically devastated site on Miyake-jima Island have their own unique physiological advantages that they can use to adapt to the harsh volcanic environment that is present on the island. On the other hand, these three pioneer species play different roles in the development and process of the Miyake-jima volcanic ecosystem (Figure 2). This is also because of their individual ecophysiological advantages that when applied together, ensure the stability of these plants during the early stages of volcanic succession and provide better conditions for the next stage of succession.

### 3.3. Is There a Correlation or a Trade-Off between Growth (A_max_ and PNUE) and Persistence (LMA)

Leaf trait relationships (e.g., LMA and A_max_, PNUE) (Figure 1) can reflect the nitrogen allocation between photosynthetic (Rubisco, light-harvesting complex) and non-photosynthetic nitrogenous compounds (cell wall structural protein) [28,46,47,48]). Numerous studies concerning leaf structure and the physiological plant responses indicated that a high LMA implied a lower PNUE and A_max_ [16,47]. In this study, the reduction of PNUE and A_max_ and the increase of LMA of *M. condensatus* was in line with other studies that have been conducted in nutrient-poor environments [16,49]. The *M. condensatus* leaves with greater LMA have a reduced PNUE because a greater ratio of the leaf N is invested in the structural proteins that comprise the cell wall. Consequently, the allocation of N for photosynthetic enzymes (e.g., Rbubisco) is reduced, resulting in a reduction of A_max_ [50,51,52]. In addition, greater LMA also causes a reduction in A_max_ through another mechanism; greater LMA involves a longer CO_2_ diffusion path from the stomata to the mesophyll cells and chloroplasts and hence reduces A_max_ [53,54]). Contrary to *M. condensatus*, the N-fixing species *A. sieboldiana* shows a different pattern, when LMA increased, the PNUE and A_max_ also increased, and this was consistent with a previous study by Choi et al. [39]. Similarly, Tang et al. [29] reported that N-fixing species *Erythrophleum fordii* had the same pattern. Therefore, it is likely that the higher leaf nitrogen content should translate into a higher photosynthetic capacity despite high LMA for N-fixing species. This is largely due to its N-fixing ability, which could provide a continuous N supply in an extremely N-limited volcanically devastated habitat. Surprisingly, for *F. japonica*, there was no significant effect of LMA on A_max_ and PNUE, which is something that has not been shown in earlier studies. This could be due to its unique N allocation pattern; *F. japonica,* which has neither a C_4_ CO_2_ fixing pathway nor the ability to continuously obtain N, allocate a fixed N content to the photosynthetic organs to enable a trade-off between growth and persistence.

### 3.4. Plant-Soil Feedback

A higher STN was found in *A. sieboldiana* and *F. japonica* than in *M. condensatus* (Table 1). The higher STN found *A. sieboldiana* might be due to its strong N fixation capacity and the maintenance of the N content stability in the leaves. However, the higher N found in and under *F. japonica* is more difficult to interpret. It appears that *F. japonica* either (1) has a greater ability to acquire N through a higher root absorption capacity, demonstrating greater root proliferation, or (2) colonizes uniquely fertile microsites that is it then able to maintain the fertility of. Consequently, these results suggest a strong correlation between the strategies that are employed for soil N and plant N, indicating a positive feedback cycle.

## 4. Materials and Methods

### 4.1. Study Site

This study was conducted at the active volcanic island, Miyake-jima, which is located in Japan (34°05′ N, 139°55′ E) and that covers an area of 55.44 km^2^ and has an altitude of 775.1 m in a.s.l. (Mt. Oyama). The island has a humid, temperate climate, with a mean annual temperature of 17.7 °C. The mean temperatures of the hottest and the coolest months are 26.2 °C (August) and 9.6 °C (February), respectively. The mean annual precipitation averages 2900 mm, and mean the monthly precipitation exceeds 140 mm for every month of the year [55]. The last eruption on Miyake-jima was in 2000, and this eruption destroyed a large part of the island’s ecosystem through the heavy deposition of volcanic materials including volcanic ash and through the subsequent emission of volcanic gas containing sulfur dioxide [56,57]. However, at the point of our study, there were no current effects of volcanic gas, as emissions had already ceased [55].

Based on a pre-survey in May 2016 and according to the experimental requirements, a newly created volcanically devastated site simultaneously containing three pioneer species that are typical to Miyake-jima was chosen. After the eruption in 2000, all of the original vegetation that had been previously present in the study site was completely buried by thick volcanic ash deposition. After more than 15 years of natural vegetation recovery, the vegetation coverage has significantly improved. However, at the point of our study (September 2018), the vegetation is still very sparse. The land surface is still covered with unweathered volcanic ash. The plants are completely exposed to sunlight. 

### 4.2. Plant Materials

**Figure A1:**
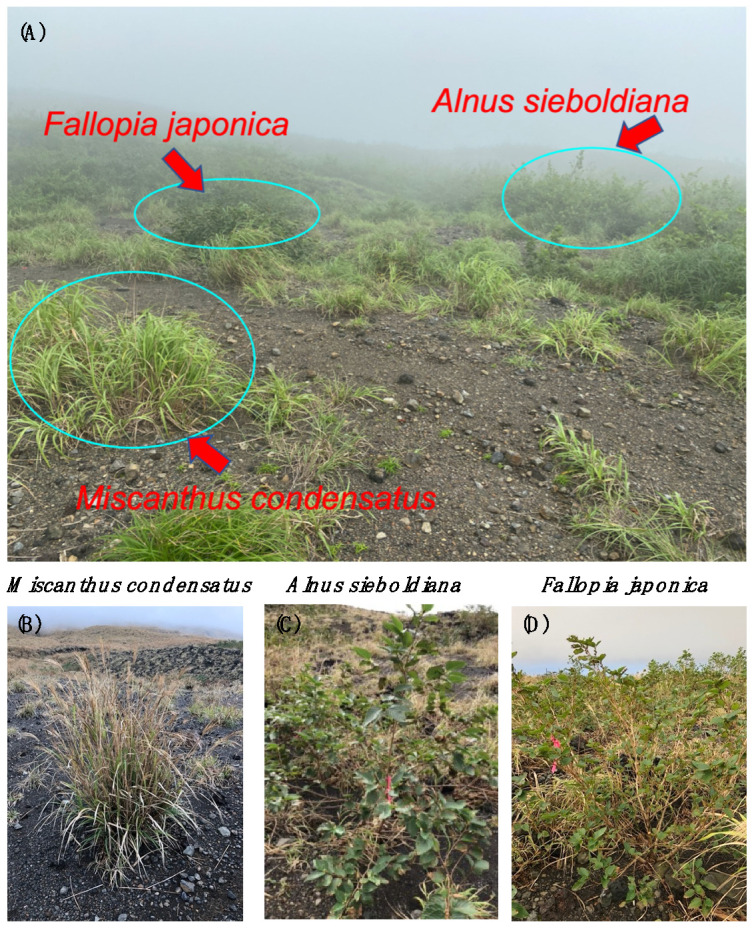
Landscape of study site (**A**) and pictures of the plants *Miscanthus condensatus* (**B**), *Aluns sieboldiana* (**C**), and *Fallopia japonica* (**D**).

In our study, we selected the three pioneer species (Figure A1 in Appendix A) that were the first invade the bare land after the eruption that took place in the year 2000. For each species, 15 individuals were selected for leaf trait measurements. The height of each species was between 0.5 and 1.5 m. The distance between the chosen individuals was at least 5 m. Sampling and measurements for each species were completed during clear, rainless days in early September 2018.

*M. condensatus* (Figure A1B), a C_4_ plant belonging to Poaceae, is one of the most typical pioneer species of volcanically devastated sites and can form dense growth; this plant is distributed across coastal areas of Japan, China, Korea, and the Pacific Islands. It is also tolerant to various environmental stresses (e.g., high salinity) [58]. On Miyake-jima Island, *M. condensatus* is also an important pioneer species and appears to have a high tolerance to SO_2_ gas and acidic soils [38]. There was a notable increase in the amount of *M. condensatus* following the eruption in 2000, and after more than a decade of change, it is still the dominant species in most locations on the island.

*A. sieboldiana* (Figure A1C) is a deciduous N-fixing pioneer tree species. In the volcanically devasted sites on Miyake-jima, its fast above-ground biomass development is due to the facilitation of the effects of N-fixation. The inorganic N soil concentration was extremely high in locations where *A. sieboldiana* was dominant. The deposition of N from *A. sieboldiana* via litterfall would decrease the soil C/N ratio, which, in turn, facilitates the net soil N mineralization and consequently provides an ample supply of inorganic N to nearby plants. The N limitation on vegetation development, which is prevalent during the early stages of succession on volcanic lava flows or similar substrates elsewhere, is thus alleviated [37].

*F. japonica* (Figure A1D) is native to Japan, Taiwan, and Korea. It is now widely naturalized in Europe and North America and is regarded as one of the worst invasive alien species. *F. japonica* has been found widely in manmade and natural habitats but is often restricted to open, sunny sites. *F. japonica* is a perennial species of the family Polygonaceae. On Miyake-jima, *F. japonica* is characterized as having thick leaves. As it has developed root systems, it is able to accumulate the necessary nutrients to ensure survival in the early stages of volcanic succession [59].

### 4.3. Field Measurement of Leaf Traits 

The photosynthetic capacity of the leaves of each species was measured using the light response curve. We measured the leaf gas exchange parameters on the newest fully expanded full-sun intact leaf using the intelligent portable photosynthesis system LCPro + (ADC BioScientific, Hoddesdon, UK). All of the measurements were performed under clear skies on sunny days in the morning between 9:00–11:00 a.m. and around solar noon at 1:00–3:00 p.m. During the measurement period, a leaf temperature of 25 °C and a CO_2_ concentration in the chamber of 420 μmol mol^−1^ were maintained. Light-response curves were determined at irradiances between 2000 and 0 µmol m^−2^ s^−1^ using a built-in LED light source in seven photosynthetic photon flux density (PPFD) steps and were fitted using a non-rectangular hyperbola model (Thornley 1976 [60]). The A_max_, DR, and LCP were calculated using light-response curves. WUE was calculated as the ratio of A_max_ to the transpiration rate (E) at PPFD saturation [61]. 

After the measurement of the gas exchange parameters, the leaves were collected to determine the leaf area (LA), (LMA), and the N_area_. The LMA (leaf dry mass/leaf area) for each individual leaf was calculated using the leaf area, as obtained by an image scanner and the leaf dry weight. To calculate leaf dry weight, the leaves were dried at 80 °C for 48 h. To calculate N_area_, the dried samples were then ground to a fine powder, and the N concentration in the leaves was measured using an NC analyzer (SUMIGRAPH NC-220F). The PNUE was calculated as the A_max_ divided by the N_area_ (PNUE, μmol CO_2_ mol N^−1^ s^−1^ = A_max_ (μmol CO_2_ m^−2^ s^−1^)/(1/14 N_area_) [50].

While measuring the gas exchange parameters, the maximum quantum yield of the photosystem II (Fv/Fm) after dark adaptation was measured using a portable Mini–PAM fluorometer (Heinz-Walz; Murchie and Lawson [62]). Prior to Fv/Fm measurement, a darkening clip was placed on the leaf for 30min to it to acclimatize to the dark. All of the measurements were performed under clear skies on sunny days in the morning between 9:00–11:00 a.m.

We estimated the Chl using a SPAD chlorophyll meter (Minolta SPAD 502 Chlorophyll Meter, Spectrum Technologies Inc., Plainfield, IL, USA), and we then used the average value to calculate the homographic model for the three pioneer species (Chl = 117.10 × SPAD/(148.84 − SPAD) [63].

### 4.4. Environmental Factors during Measurement

The photosynthetic characteristics are leaves are very sensitive to changes in the short-term environment factors. In this study, we recorded the environmental data of each measurement to determine the response of the leaf characteristics to environmental factors.

To assess the effect of rainfall on the leaf traits, the rainfall distribution for the week before the measurement day was analyzed. We calculated the integrated rainfall from before the photosynthetic measurements, which were taken from the daily rainfall data from the Japan Meteorological Agency [56]. We calculated the integrated rainfall within 3 days before each measurement and examined the effects of the rainfall on the leaf traits of each species. 

After measuring the leaf traits, soil samples from under the measured plants were collected using 100 mL core samplers that were 5 cm in depth to determine the soil properties for each species. Three cores were sampled from the soil around the root crown (approximately 10 cm away from the root crown) and were subsequently combined and mixed. After being air-dried, the samples were passed through a 2 mm sieve, and the roots were removed. The soil total C (STC) and STN were analyzed with SUMIGRAPH NC-220F, using samples smaller than 0.05 mm.

Hemispherical photographs were taken using a Nikon Coolpix 990 and a Nikon FC-E8 Fisheye Converter (Nikon, Tokyo, Japan) for every leaf physiological trait measurement. Photographs were taken above each plant. The photos were analyzed to calculate the canopy openness. The CO was defined as the fraction of open sky in the hemisphere that was visible from a point beneath the canopy and was used as an index of the light availability experienced by each individual species. 

### 4.5. Data Analysis

All of the statistical analyses were performed in the R Environment [64]. We compared the means of each leaf trait by one-way ANOVA with a post hoc Tukey HSD test. All of the leaf traits data were log10-transformed to fit a normal distribution and to meet the parametric assumption of homogeneity of variance (using Shapiro–Wilk and Bartlett tests) (Table 3). We used a regression analysis to test the relationship between LMA with A_max_, PNUE and N_area_ with A_max_, and PNUE (Figure 1). 

## 5. Conclusions

We conclude that pioneer species differing in N-fixing ability and photosynthetic pathways have their individual ecophysiological advantages, N trade-off patterns, and plant–environment relationships that are used by these species as a stress tolerance strategy in volcanic habitats with N limitations. The growth advantages that are associated with these ecophysiological leaf traits can be distinguished by the ecological characteristics of each species. *M. condensatus* shows the highest leaf photosynthesis rates. The higher rates were partially a result of higher water and nitrogen use efficiencies. The leaf nitrogen use efficiency in *M. condensatus* appears to be high, even for a C_4_ grass. *A. sieboldiana* relies on its N-fixing ability and has a higher N_area_ to compensate for its photosynthetic capacity. Regarding *F. japonica*, its higher LMA, Chl, Fv/Fm demonstrates its better ability to capture light. The divergence among the trait responses of the ecological characteristics of these different species that are associated with ecological strategies reflect their ability to adapt to the harsh volcanic environment. The diverse adaptation pattern among suites of traits may also promote the coexistence of species across the volcanic environment by increasing niche differentiation or by generating competitive trade-offs between species.

## Figures and Tables

**Table 1:** Location and growth environment of each pioneer species in study site. Different letters indicate significant differences between the pioneer species revealed by Tukey’s post hoc test at a significance level *p* < 0.05 after one-way ANOVA. Abbreviations are meters above sea level (m a.s.l); canopy openness (CO) (*n* = 15); air temperature (AT); soil total carbon (STC) (*n* = 15); total soil N (STN) (*n* = 15).

Factor	*A. sieboldiana*	*F. japonica*	*M. condensatus*
Location	34°04.689′ N, 139°30.816′ E		
Altitude (m a.s.l)	500		
Ash depth (cm)	30–35		
CO (%)	80.51 ± 0.41 a	80.64 ± 0.27 a	80.48 ± 0.34 a
Precipitation (mm)	42.23 ± 3.07 a	41.05 ± 0.64 a	40.72 ± 0.11 a
AT (°C)	27.23	26.49	26.54
STC (%)	0.30 ± 0.02 a	0.24 ± 0.02 ab	0.22 ± 0.03 b
STN (%)	0.04 ± 0.0008 a	0.03 ± 0.0016 a	0.02 ± 0.0027 b

**Table 2:** List of leaf traits measured in Miyake-jima and categorized according to their function.

Trait Name	Abbreviation	Units	Trait Functions
Leaf area	LA	cm^2^	Photosynthetic capacity, light interception
Leaf mass per area	LMA	g m^−2^	Photosynthetic capacity, defense, adaption to light
Nitrogen concentration per leaf area	N_area_	g m^−2^	Photosynthetic capacity, palatability, decomposability
Maximum quantum yield of Photosystem II	Fv/Fm	-	Photosynthetic capacity, photodamage, capacity to dissipate light energy, responsiveness to light quality
Chlorophyll content	Chl	µg cm^−2^	Photosynthetic capacity, light interception
Light-saturated photosynthetic rate	A_max_	µmol CO_2_ m^−2^ s^−1^	Metabolic capacity
Photosynthetic N-use efficiency	PNUE	μmol CO_2_ mol N^−1^ s^−1^	Balance nitrogen allocation
Transpiration rate	E	mmol^−1^ H_2_O m^−2^ s^−1^	Water use capacity, metabolic capacity
Light compensation point	LCP	µmol m^−2^ s^−1^	Light use capacity
Dark respiration	DR	µmol m^−2^ s^−1^	Metabolic capacity
Water use efficiency	WUE	μmol CO_2_ mmol^−1^ H_2_O	Water use capacity

**Table 3:** Mean (±SE, *n* = 15) leaf trait values of three pioneer species in the volcanically devastated site in Miyake-jima. Data analyzed using one-way ANOVA with a post hoc Tukey HSD test. All variables were log10-transformed prior to analysis. Significant differences at *p* < 0.05 among species are shown in different letters. Full names of leaf traits are given in Table 2.

Leaf Traits	*A. sieboldiana*	*F. japonica*	*M. condensatus*	*F*	df	*p*
LA	42.30 ± 2.03 a	42.49 ± 3.07 a	54.13 ± 2.48 b	6.584	2	**<0.05**
LMA	101.33 ± 4.96 a	117.59 ± 5.60 b	98.99 ± 2.48 a	4.319	2	**<0.05**
N_area_	2.55 ± 0.06 a	1.96 ± 0.07 b	1.23 ± 0.07 c	73.68	2	**<0.001**
Fv/Fm	0.77 ± 0.003 a	0.83 ± 0.001 b	0.71 ± 0.008 c	115.4	2	**<0.001**
Chl	40.39 ± 1.19 a	49.82 ± 1.38 b	25.05 ± 0.48 c	171.9	2	**<0.001**
A_max_	14.21 ± 0.71 a	13.58 ± 0.78 a	18.48 ± 0.93 b	8.988	2	**<0.001**
PNUE	79.16 ± 4.91 a	98.13 ± 5.10 b	221.47 ± 16.83 c	68.6	2	**<0.001**
E	2.94 ± 0.37 a	3.57 ± 0.24 a	1.53 ± 0.12 b	16.27	2	**<0.001**
LCP	34.06 ± 2.28 a	29.66 ± 2.31 a	35.72 ± 3.15 a	0.527	2	0.594
DR	1.34 ± 0.10 a	1.06 ± 0.06 b	0.91 ± 0.07 c	6.941	2	**<0.01**
WUE	5.89 ± 0.69 a	4.03 ± 0.28 b	13.71 ± 1.69 c	43.75	2	**<0.001**

## Appendix A

**Table A1:** Comparison of leaf trait values (mean ± SE) among the three pioneer species (*n* = 15 for each species) (this study) collected from the volcanically devastated site on Miyake-jima Island with the data of same functional type leaves from the GLOPNET dataset (range and mean ± SE) (Wright et al., 2004) and the leaf traits values (mean ± SE) of other pioneer species living in similar habitat conditions. Life form, grass (G), tree (T), herb (H); N-fixing ability, yes (Y), no (N); photosynthetic pathway, C_3_ and C_4_. Traits, N_area_, leaf nitrogen content per area (g·m^−2^); A_max_, light saturated photosynthetic rate per leaf area (µmol m^−2^ s^−1^); PNUE, photosynthetic N use efficiency (µmol CO_2_ mol N^−1^·s^−1^). Shaded cells were used to improve the table readability.

Specie/Family/Plant Type/N-Fixing Ability/Photosynthetic Pathway	N_area_	A_max_	PNUE	Site	Reference
*Miscanthus condensatus* (Poaceae) (G) (N) (C_4_)	1.23 ± 0.07	18.48 ± 0.93	221.47 ± 16.83	Miyakejima Island	This study
*Fallopia japonica var. hachidyoensis* (Polygonaceae) (H) (N) (C_3_)	1.96 ± 0.07	13.58 ± 0.78	98.13 ± 5.10		This study
*Alnus sieboldiana* (Betulaceae) (T) (Y) (C_3_)	2.55 ± 0.06	14.21 ± 0.71	79.16 ± 4.91		This study
Plant functional type					
Grass C_4_ non-N-fixing (16)	1.39 ± 0.24	20.88 ± 1.72	241.32 ± 27.15	GLOPNET dataset	Wright et al., 2004
	0.53–3.39	13.04–31.70	53.87–357.10		Wright et al., 2004
Herb C_3_ non-N-fixing (205)	1.55 ± 0.05	14.71 ± 0.56	132.85 ± 5.35		Wright et al., 2004
	0.26–5.29	1.00–28.68	27.70–281.41		Wright et al., 2004
Tree C_3_ N-fixing (66)	2.54 ± 0.22	13.24 ± 1.10	73.72 ± 6.51		Wright et al., 2004
	0.91–5.44	3.67–36.60	16.33–162.427		Wright et al., 2004
Pioneer species in N low habitats					
*Rhynchelytrum repens* (Poaceae) (G) (N) (C_4_)	0.67	21.40	447.16	Hawaiian volcanic	Funk and Vitousek 2007
*Heteropogon contortus* (Poaceae) (G) (N) (C_4_)	0.78	19.20	344.62		Funk and Vitousek 2007
*Paspalum urvillei* (Poaceae) (G) (N) (C_4_)	0.77	21.40	389.09		Funk and Vitousek 2007
*Eragrostis variabilis* (Poaceae) (G) (N) (C_4_)	0.97	13.60	196.29		Funk and Vitousek 2007
*Holcus lanatus* (Poaceae) (G) (N) (C_3_)	1.09	8.20	105.32		Funk and Vitousek 2007
*Deschampsia nubigena* (Poaceae) (G) (N) (C_3_)	2.22	11.20	70.63		Funk and Vitousek 2007
*Metrosideros polymorpha (Gaud.)* (Myrtaceae) (T) (N) (C_3_)	2.46	8.83	38.60	Hawaiian volcanic	Cordell et al., 2001
*Ageratina riparia* (Asteraceae) (T) (N) (C_3_)	1.23	12.70	144.55	Hawaiian volcanic	Funk and Vitousek 2007
*Dubautia scabra* (Asteraceae) (T) (N) (C_3_)	1.06	10.50	138.68		Funk and Vitousek 2007
*Desmodium sandwicense* (Fabaceae) (T) (N) (C_3_)	1.91	6.20	45.45		Funk and Vitousek 2007
*Sesbania tomentosa* (Fabaceae) (T) (N) (C_3_)	2.12	9.50	62.74		Funk and Vitousek 2007
*Pyracantha angustifolia* (Rosaceae) (T) (N) (C_3_)	2.48	20.70	116.85		Funk and Vitousek 2007
*Osteomeles anthyllidifolia* (Rosaceae) (T) (N) (C_3_)	1.19	13.80	162.35		Funk and Vitousek 2007
*Psidium cattleianum* (Myrtaceae) (T) (N) (C_3_)	1.93	6.70	48.60		Funk and Vitousek 2007
*Metrosideros polymorpha* (Myrtaceae) (T) (N) (C_3_)	1.63	2.60	22.33		Funk and Vitousek 2007
*Templetonia retusa* (Vent.) R.Br. (Fabaceae) (T) (N) (C_3_)	2.94	13.70	67.20	Australia sand dune	Guilherme Pereira et al., 2019
*Acacia rostellifera* Benth. (Fabaceae) (T) (N) (C_3_)	2.68	17.80	121.80		Guilherme Pereira et al., 2019
*Anthocercis littorea* Labill. (Solanaceae) (T) (N) (C_3_)	2.55	13.90	81.20		Guilherme Pereira et al., 2019
*Dioscorea hastifolia* Endl. (Dioscoreaceae) (T) (N) (C_3_)	1.54	11.30	103.60		Guilherme Pereira et al., 2019
*Myoporum insulare* R.Br. (Scrophulariaceae) (T) (N) (C_3_)	2.44	14.50	84.00		Guilherme Pereira et al., 2019
*Spyridium globulosum* (Labill.) Benth. (Rhamnaceae) (T) (N) (C_3_)	1.98	17.00	120.40		Guilherme Pereira et al., 2019
*Aristotelia serrata* (Elaeocarpaceae) (T) (N) (C_3_)	1.54	12.10	110.23	New Zealand	Atkin et al., 2013
*Griselinia littoralis* (Griseliniaceae) (T) (N) (C_3_)	2.59	8.80	47.41		Atkin et al., 2013
*Hebe salicifolia* (Plantaginaceae) (T) (N) (C_3_)	2.35	15.80	52.98		Atkin et al., 2013
*Olearia avicenniifolia* (Asteraceae) (T) (N) (C_3_)	2.28	17.00	49.75		Atkin et al., 2013
*Coriaria arborea* (Coriariaceae) (T) (Y) (C_3_)	2.77	9.50	47.99		Atkin et al., 2013
*Conyza canadensis* (Asteraceae) (H) (N) (C_3_)	1.47	17.00	161.90	Hawaiian volcanic	Funk and Vitousek 2007
*Pseudognaphalium sandwicensium* (Asteraceae) (H) (N) (C_3_)	0.97	8.00	115.46		Funk and Vitousek 2007
*Hypochoeris radicata* (Asteraceae) (H) (N) (C_3_)	1.03	14.50	197.09		Funk and Vitousek 2007
*Argyroxiphium kauense* (Asteraceae) (H) (N) (C_3_)	1.66	9.10	76.75		Funk and Vitousek 2007
*Plantago lanceolata* (Plantaginaceae) (H) (N) (C_3_)	1.76	14.40	114.55		Funk and Vitousek 2007
*Plantago hawaiensis* (Plantaginaceae) (H) (N) (C_3_)	1.41	10.30	102.27		Funk and Vitousek 2007
*Olearia axillaris* (DC.) Benth. (Asteraceae) (H) (N) (C_3_)	2.87	13.70	70.00	Australia	Guilherme Pereira et al., 2019
*Aconogonum weyrichii* (Polygonaceae) (H) (N) (C_3_)	1.22	22.70	159.16	Japan mount Fuji	Sakata et al., 2006
*Reynoutria japonica* (Polygonaceae) (H) (N) (C_3_)	1.58	18.60	162.75		Sakata et al., 2006
